# Strategies to reduce the cancer burden and improve access to effective and affordable cancer interventions in Europe

**DOI:** 10.1002/1878-0261.70058

**Published:** 2025-05-21

**Authors:** Anton Berns, Ulrik Ringborg, Julio Celis, Bengt Jönsson, Michael Baumann

**Affiliations:** ^1^ European Academy of Cancer Sciences Stockholm Sweden; ^2^ Division of Molecular Genetics The Netherlands Cancer Institute Amsterdam The Netherlands; ^3^ Cancer Center Karolinska Karolinska University Hospital Solna Stockholm Sweden; ^4^ Department of Economics Stockholm School of Economics Sweden; ^5^ German Cancer Research Center (DKFZ) Heidelberg Germany

**Keywords:** affordability, comprehensive cancer centre, EU Cancer Mission, funding, health‐related quality of life, PI‐initiated clinical trials

## Abstract

The development of new anticancer treatments, their clinical evaluation and introduction into the healthcare system need improvement. New drugs and cell therapies often come with significant costs for society while only marginally improving patients' survival and health‐related quality‐of‐life. Therefore, bold, innovative clinical trials with critical assessment of the efficacy and cost‐effectiveness of new preventive measures and medical treatments are needed to ensure that patients and society benefit. Drug development programmes controlled by pharma should be complemented with initiatives such as stop studies, dose reduction, combination and repurposing trials. These should be validated in academia‐initiated trials supported by societal funds. Special attention should be devoted to paediatric and rare adult cancers. Comprehensive Cancer Centres (CCCs) covering the entire cancer research continuum, present throughout the EU, are critical for this. More of such centres must be established concomitantly with a robust accreditation methodology to ensure that they meet appropriate quality standards. It is crucial that funding for these initiatives, now temporarily and partially provided by the EU Cancer Mission and Europe's Beating Cancer Plan, is secured for a much longer period.

## Graphical abstract

Comprehensive cancer centre (CCCs) and CCCs of Excellence (CCCoE) integration in healthcare. Through outreach to surrounding community hospitals, CCCs enable wider access to top‐clinical cancer treatments and care, thereby facilitating the swift enrolment of patients into data‐rich clinical trials (PI‐initiated trials testing new concepts, drug combination trials and dose reduction trials) and outcomes research (assessing real‐world effectiveness and health economics of new treatments).

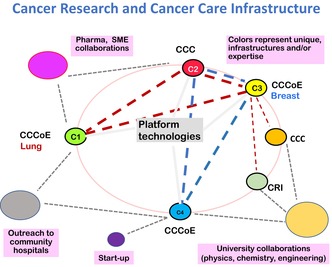

**Abbreviations**
CCC: Comprehensive Cancer Centre (integrated basic, translational and clinical research)
CCCoE: Comprehensive Cancer Centre of Excellence
CraNE: Joint Action to establish an EU network of Comprehensive Cancer Centres
EACS: European Academy of Cancer Sciences
EBCP: Europe's Beating Cancer Plan
EMA: European Medicines Authority
EU: European Union
EUnetCCC: European Network of Comprehensive Cancer Centres
FDA: Food and Drug Administration
HRQoL: health‐related quality of life
MoC: EU Mission on Cancer
OECI: Organisation of European Cancer Institutes
PFS: progression free survival
QALY: quality adjusted life years
SPC: supplementary protection certificate

## 1 Introduction

The European Academy of Cancer Sciences (EACS), a community of cancer experts, organised a one‐day meeting with eight European cancer charities, representing a network for funding cancer research with direct links to research institutes, ministries, policymakers and patient organisations at the Member State level, to discuss shortcomings in the current cancer research landscape in the European Union (EU) and how these might be tackled. Cancer is a rising problem in the EU as well as globally, with increasing challenges to effectively translate new biological knowledge into better treatments and make these widely accessible to cancer patients at affordable prices [1]. New insights mostly result from academic public research, which is then translated into treatments in which new drugs and therewith pharmaceutical companies play an increasingly prominent role. Unfortunately, this ecosystem does not function properly [2] and needs an overhaul to assure that new treatments benefit patients at affordable costs.

Europe's Beating Cancer Plan (EBCP) and the EU Mission on Cancer (MoC) aim to make high‐quality cancer care accessible and affordable for all EU citizens [3]. Investments in the complete cancer research trajectory and closer integration with cancer treatment and care throughout Europe are needed for this. Several critical questions remain on how to achieve this (Box 1).

Box 1 **The Questions**

How can we effectively support research covering the entire cancer research continuum from basic to clinical to outcomes and health economics research?How can we promote robust health economics studies to inform policies for cost‐effective pricing of new cancer medicines, for reducing inequalities and for making therapeutics/cancer care affordable for all?How to make more effective use of available resources for cancer research to reduce cancer incidence, increase cure rates, improve patients' Health‐Related Quality of Life (HRQoL) and palliative care?How to set up infrastructures needed to optimally facilitate development, evaluation, and implementation of new treatment paradigms?

This opinion article encapsulates *the key issues* that were explored during the EACS consulting meeting with eight European charities. The focus was on how the EACS, as one of the key players in the field, could bolster joint cancer research initiatives in Europe. This discussion, which prompted the authors to write this opinion document, outlines the EACS's perspective on addressing the challenges we face and leveraging the opportunities that arise from the MoC and EBCP initiatives. The intention is to use this document as a foundation for further discussions, highlighting the collaborative nature of these initiatives and the collective effort involving charities, other funding bodies, and policymakers.

## 2 Issues at stake and consequences

Issues that are often neglected need to be considered in the context of the increasing number of new cancer cases, the even faster‐growing number of patients living with a cancer diagnosis and the rapid development and introduction of new treatments for cancer are listed in Table 1.

**Table 1:** Issues at stake and consequences.

Primary, secondary, and tertiary prevention	Although it can massively contribute to the long‐term goals to reduce the suffering from cancer, prevention receives insufficient emphasis. More priority needs to be given to primary prevention in order to reduce exposure to cancer inducing agents, to discourage lifestyles that promote cancer development or mitigate its consequences (e.g. vaccination), to secondary prevention through focused screening programmes (e.g. for prostate, ovary, lung and pancreas cancer) and to tertiary prevention by measures preventing complications once cancer is detected and treated
Assessment of novel anticancer treatments	There is a widely felt concern about the present assessment of new anticancer treatments in clinical trials and their acceptance as standard treatment, with too much emphasis on progression‐free survival (PFS) and overall survival (OS), often presented by immature data or reported using surrogate markers as response parameter. These are frequently assessed in small clinical trials, often single arm, and through subgroup analyses [4, 5]. In addition, QALY (Quality‐Adjusted Life Years gained) needs to become the standard outcome parameter. Advancing clinical trial methodology and health economics research on drug development processes are also important for pricing and reimbursement decisions [6]
Follow‐up monitoring	Often there is a lack of follow‐up monitoring in assessing the benefits of new interventions compared to standard of care in real‐world conditions. This has become an even more critical issue due to fast‐track drug registration and the increasing number of cancer medicines on the market
Treatment duration and dosing	Insufficient attention is devoted to optimal dosing and the required duration of interventions in connection with the achieved patient benefits and costs
Rare and paediatric cancers	The development and testing of new interventions for paediatric and rare adult cancers is lagging behind
Established treatment regiments	It is difficult to obtain support for trials that do not explore new drug regimens, such as new surgical and radiotherapeutic interventions
Clinical trial redundancy	There is too much redundancy in clinical trials that are testing the efficacy of similar drugs and biologicals (‘me‐too drugs’) [7]. Objective arguments are needed to justify the evaluation of large numbers of drugs/biologicals against the same targets without convincing indications that these are more effective or have less toxic side effects. Furthermore, ‘Me Too’ clinical trials hamper innovation, reduce patient access and increase cost
Testing of novel treatment concepts by scientific research	High‐quality academic research is critical for the development of new treatment paradigms. However, it is facing difficulties in exploring new concepts such as the testing of specific drug combinations, alternative scheduling, treatment at an earlier stage of disseminated disease, adaptive medical treatment or repurposing of drugs, due to poor drug access and/or financial support for conducting such trials
Registration of novel treatments	The registration process (FDA/EMA) is unnecessarily bureaucratic, making it particularly cumbersome (and costly) for academia and small biotech to register new drugs/biologicals. However, the recent opportunity to register available medications based on a new rule (rule 48) of the General Pharmaceutical Legislation, which would allow not‐for‐profit entities to submit clinical evidence for a new therapeutic indication that is expected to fulfil an unmet need, is an improvement
Pharmaceutical industry	Big pharmaceutical companies have an excessively dominant role in the clinical trial‐arena. They are by far the most prominent funder of translational and clinical cancer research providing more than three‐quarters of the total budget in both Europe and the United States. They spend over 10‐fold more on clinical trials than is provided by public sources [8, 9]. This makes academic centres and physicians overseeing the trials too dependent on pharma, not only for conducting the trials but also for maintaining the infrastructure required for running such trials

The issues introduced above in Table 1 have resulted in a situation in which we insufficiently capitalise on the potential of academic translational research, the catalyst for new paradigms in prevention, early detection and treatment/care of cancer. This restrains the development of new affordable treatments, effective drugs, biologicals and cell therapies for patients. This situation is further exacerbated by inadequacies in the following issues:

- **Patent assignment and protection issues**: Patent rights are often transferred from academia to pharmaceutical companies without clear binding agreements for further development and pricing. Patent protection is subsequently too exclusively exploited for maximising financial benefit rather than exploiting the potential of the invention to serve patients.
- **Patient representatives**: Involvement of patient experts in clinical research is underdeveloped in Europe. Next to being informed about the type of research and its goals, patients need to be more closely involved in defining the questions to be addressed in research projects and the route along which these will be answered in preclinical model systems and clinical trials.
- **Unreasonably high pricing of anticancer drugs**: Prices are insufficiently driven down by competition from similar ‘Me Too’ drugs as one would expect. Market forces appear not to function properly. The obligatory disclosure of pricing should be considered.
- **Production of new generation anticancer drugs**: The nature of new generations of drugs has changed due to the complexity of producing them and assessing their quality. Once off‐patent, production is less easily taken over by drug companies focussed on ‘Generics’, which is particularly the case for biologicals. This enables high pricing for many anticancer agents for extended periods, often supported by Supplementary Protection Certificates (SPC). European public health insurers and healthcare payers should jointly negotiate a justifiable pricing, thus making room in the budgets for a more rapid introduction of new promising alternatives.
- **Access to state‐of‐the‐art treatments**: Cancer patients deserve state‐of‐the‐art treatments, and society should be able to offer those. However, there are growing inequalities in access to new cancer treatments between countries with high and low incomes in Europe [10, 11] due to the high pricing of drugs. Even rich countries will soon be unable to afford the skyrocketing costs of precision medicine drugs [12], biologicals and cell therapies. They will not even be able to make informed choices between the rapidly increasing number of alternatives available for spending the limited public budgets. Private payment for new cancer medicines is not an option for most cancer patients.

It is particularly important to timely address these issues in the context of the MoC and the EBCP, although it is important to realise that the funding for these programmes is only around 2% of what is spent on cancer research and around 1% of the total spent on cancer medicines in Europe. Besides the need to significantly increase the public funding for cancer research, in particular clinical trials that compare relevant treatment options and provide reliable outcomes, policies must be developed to reform the entire cancer research market, private and public; this should include expanding the role of EMA to take greater responsibility in defining the relevant outcome studies to be conducted during the development process. The two EU initiatives (EBCP and MoC) provide valuable incentives to boost expertise and stimulate collaboration. The goal to establish an array of networks of Comprehensive Cancer Centres (CCCs) that offer state‐of‐the‐art treatments to patients [13], concurrent with conducting innovative translational research, including clinical trials, is an important asset. However, there are several issues related to CCCs that need attention:

- **Agreement on an unequivocal definition of a CCC**. The OECI, German Cancer Aid, CraNE, EUnetCCC and EACS should prioritise reaching a consensus on the CCC definition and the qualifications a CCC needs to meet.
- **A shared accreditation methodology for CCCs**. that clearly states what is required to qualify as a CCC. It is also worth considering to what extent the accreditation parameters can be aligned with accreditation systems already in place in the different Member States. For instance, Germany already had a CCC accreditation programme for over 15 years [14], which has been the basis for successfully structuring translational cancer research nationally. There are at present discussions between OECI and German Cancer Aid about the harmonisation of the two accreditation methodologies, including how to enable quality assurance of the CCC ‘outreach area’ that would allow all patients in the EU to benefit from the two EU initiatives once the aims are reduced to practice (see below). In addition, specific quality parameters must be defined for CCCs that opt for additional distinctions of excellence in specific expertise areas [15]. These latter centres (CCCoE) will be best positioned to develop new concepts and focus on practice‐changing clinical trials [16].
- **Growth in the number of CCCs**, especially in countries with lower income per capita and thus fewer resources for cancer research, is crucial. Incentives need to be put in place to build such centres. Equally crucial is the cross‐border twinning with high‐quality established centres, which is an appealing option and a source of reassurance. Cross‐border services provided by established CCCs to countries or regions of countries with sparse populations should be considered. The consensus is that over 100 CCCs are required in the EU to serve the population (with at least 1 CCC per country and around 1 CCC per 4–5 million citizens). This was the goal of the CraNE Joint Action Plan and is now further pursued in the EUnetCCC project. However, it will take many years beyond the EBCP and the MoC timeframe to accomplish this, and therefore, continued funding is required. The same holds for the endowment of networks of CCCs and long‐term educational support. This has to come from new funding sources.
- **More support for innovative investigator‐initiated clinical trials**. Many academic centres are set up to increase their income by conducting industry‐sponsored trials. While industry and academia must work together, there is also a need for academia, healthcare providers and insurers, including reimbursement authorities, to influence the direction and content of these trials. If the main purpose is to generate income for the centre, we may go in the wrong direction. The academic trial portfolio needs to be bold and include, next to the testing of new innovative concepts underscored by strong preclinical data, prevention trials, repurposing trials, drug combination trials and trials in paediatric and rare adult cancers. Academic clinical trials are also of key importance to assess new methods, including imaging, radiotherapy and surgical approaches, and supportive care strategies, as all of these are largely neglected by industry. These trials are crucial for enabling rational decisions on appropriate and cost‐effective cancer treatments, including palliative care, providing assurance about the effectiveness of the treatments.
- **Placing academia more clearly in the driver's seat of clinical trials** with the accompanying preservation of intellectual property rights (and proper arrangements for individual inventors) is needed to guarantee more control over the development process and subsequent use and pricing of new interventions. Exploring the effects of reduced drug dosage, alternative scheduling schemes without affecting response rates and identifying biomarkers for response and adverse events are particularly relevant and should have a high priority. Next, to mitigate side effects, they will also reduce treatment costs. Given the hesitation from the pharmaceutical industry to support such trials and the lack of funding by regular governmental sources, charities have started, in some instances with support from health insurers, to jointly fund dose reduction trials and registration trials, the latter being required for offering an intervention as part of treatment options to patients. Results from some of these trials show that much can be gained by this strategy, both regarding improved HRQoL for patients as well as cost‐effectiveness (Box 2). However, given the high costs, current public funding streams are unable to cover the actual need to conduct such trials. This further illustrates the importance that rational arguments considering effectiveness, side effects and costs of treatments become an obligatory part of every drug assessment scheme during its development trajectory.

Box 2 **Prevention**

Prevention must become a major component to reduce the suffering from cancer. As around 50% of cancers are preventable, it deserves much more emphasis. Currently, only 7% of cancer research funding is devoted to prevention. Research into new causes of cancer, identification of new genetic predisposing factors and a concerted effort to reduce exposure to known risk factors in a cost‐effective way is needed, for example by taxation, legislation, promoting protective measures (e.g. HPV vaccination, sun protection) or combinations of these. One fast‐track route is persuading regulatory agencies to implement measures that have been successfully applied in other countries, as well as stimulating implementation research, for example how to motivate citizens to abandon unhealthy lifestyles.

- **The integration of outcomes research into the drug assessment process** is crucial, since a significant portion of 150 anticancer drugs introduced in the last 15 years shows only modest improvements over standard therapy [2, 17, 18], and yet very substantially contribute to the rising healthcare costs associated with cancer treatments [11, 19]. This does not jeopardise swift conditional approval of treatments if it is paired with the requirement for comprehensive follow‐up evaluation in meticulously conducted real‐world settings [20] before long‐term market access is granted and pricing is agreed upon. The current inadequacy of postmarketing studies and real‐world data for clinical effectiveness assessment [21] needs to be replaced by solid outcomes research, combined with proper health economic assessment, thereby providing perspectives for significant improvement in cancer treatments.
- **Assessing evidence‐based protocols for treating patients with uncurable disease** is difficult due to the increasing number of new anticancer agents reaching the market. Although good guidelines for primary treatment of most cancers are available and regularly updated, evidence‐based guidelines for the treatment of patients with advanced disseminated disease or in need of early palliative care are largely lacking. HRQoL support is the main goal for this group of patients. Unfortunately, new anticancer agents are not assessed for clinical effectiveness in this patient group and may actually compromise their HRQoL without improving survival.
- **Prevention must become a major component of the efforts to reduce the suffering from cancer**. As for up to 50% of cancers, the causes are well‐known; governments have to be mobilised to introduce legislature to mitigate exposure to agents that promote cancer development (e.g. exposure to UV and toxic substances, use of tobacco, vaping, alcohol, sugar‐containing soft‐drinks). It is also essential to motivate citizens to pursue a healthy lifestyle and take advantage of prevention options (e.g. HPV vaccination). This public motivation is a key factor in cancer prevention, requiring input from behavioural sciences. In addition, evidence‐based approaches in situational prevention, such as taxation or banning policies, need to be pursued. Also, more research into environmental causes of cancer and identification of new genetic predisposing factors should be accompanied by a concerted effort to mitigate new risks identified in prevention research efforts (an illuminating recent example is the lung cancer‐promoting effect of small particle matter) [22].
- **Early detection deserves more emphasis**. We need better and more reliable methods to identify individuals with early disease as well as personalised, risk‐adapted early detection strategies. This requires better insight into the biology of cancer initiation and the genetic and environmental factors that increase the risk of developing tumours. Population screening methods must meet stringent criteria such as high effectiveness, minimal impact on HRQoL and cost‐effectiveness. Overall, it will be important to take advantage of insights acquired in psychosocial and economic sciences in cancer prevention and treatment.

## 3 Proposed initiatives

The EBCP project EUnetCCC is expected to set the stage for the establishment of robust quality assurance programmes for CCCs and CCCoE, which play a pivotal role in guaranteeing continuous innovation in cancer health care as they will provide the necessary infrastructure and critical mass of professionals needed for advanced translational cancer research and assure that prevention research received adequate attention (Box 3). The involvement of the outreach areas of the CCC will make high‐quality cancer health care available for all patients. In addition, access to a large patient population and biological materials offers exciting new research opportunities. Additional questions that must be dealt with are how governance of CCCs and consortia thereof have to be secured. In this regard, CCCs in the United States have served as an example of how national agencies such as the National Cancer Institute can promote CCC quality, local outreach and collaboration by incentivising funding.

Box 3 **Scheduling**

Primary outcome analysis of the phase 3 SONIA trial (BOOG 2017‐03) on selecting the optimal schedule for the use of cyclin‐dependent kinases 4 and 6 (CDK4/6) inhibitors for patients with hormone receptor‐positive (HR^+^), HER2‐negative (HER2^−^) advanced breast cancer (ABC). It appeared that first‐line use of CDK4/6i + ET does not provide statistically significant nor clinically meaningful PFS benefit compared to second‐line use in women with HR^+^, HER2^−^ ABC. Use in first‐line prolongs the time on CDK4/6i by 16.6 months and increases toxicity and costs [25].

The research portfolio to address these issues must be effectively coordinated and funded. This requires knowledgeable and coordinated funding organisations with sufficiently deep pockets. Several options come to mind:

Establishing a ‘European Cancer Agency’, was first discussed in the EU project EUROCAN+Plus 2005–07 in terms of a European Cancer Institute or a European Cancer Initiative, which addressed governance and funding issues [14]. One might envision an intergovernmental organisation that receives funding from healthcare providers, as well as governmental funding organisations and private parties to finance research throughout the cancer research continuum. Such an agency could provide funding for establishing CCCs in underprivileged areas, support the twinning and networks of CCCs and oversee their accreditation. It could offer research grants (using ERC‐like principles for funding preclinical‐translational, clinical and outcome research), take responsibility for EU‐wide patient‐related databases and repositories and secure GDPR‐proof data access. It could also coordinate the funding of academia‐led clinical registration trials next to serving as an intermediate entity to ensure access to a portfolio of drugs to facilitate combination and new drug‐scheduling trials. In addition, it might develop guidelines concerning drug patent ownership and fair pricing for academia and pharmaceutical industry.

Health Insurers and publicly funded healthcare systems in Europe are in an excellent position to support academia‐led clinical trials. With their responsibility for patients and access to records, they could provide a parallel track to pharma‐sponsored trials and are particularly well‐positioned to support trials for which academia holds the intellectual property rights, for drug repurposing, drug combination trials, rescheduling trials and exploration of new biomarkers. Health insurers are also in a good position to support registration trials of already certified drugs for new indications, an issue subject to Article 48 discussed in the EU. Given that the effects of lower dosing and shorter administration times are poorly explored territory, this is a particularly important area to reduce side effects, and it might even reduce the costs of drugs during the execution of such trials (see example of the SONIA trial Box 3). Also, highly individualised treatments, such as cell and gene therapies, are, given the extremely high cost charged by pharma for these individualised treatments, well‐suited for academic development and can be produced cost‐effectively in an academic hospital setting [23, 24]. If health insurers would dedicate just a few percent of their current expenditure for expensive anticancer agents to this academic clinical trial activity that should include development and evaluation of new surgical and radiotherapy procedures, substantial progress could be made. This would well align with their mission as public healthcare providers have a strong interest in relevant data for their decisions on pricing, reimbursement and procurement. It would not only benefit patients; it would substantially reduce the cost spent on anticancer treatments (Box 3).

## 4 Conclusions

The charities and EACS, each having patient organisations as critical constituents of their organisations, with broad societal representation, should join forces and use their societal influence in the EU and their respective Member States to incentivise politicians, governmental health agencies and healthcare insurers to provide long‐term support for these initiatives by establishing a joint fund, with an expert board and review panels to decide on applications (ERC‐like arrangement). This will benefit patients as well as society at large. In our view, this would constitute a promising route to make new and more effective anticancer treatments accessible and affordable for those in need, thereby bringing equal access [26] to state‐of‐the‐art cancer treatments in closer sight. Similar initiatives should also be encouraged in other areas of the world.

## Conflict of interest

The authors declare no conflict of interest.

## Author contributions

AB drafted the initial manuscript that was complemented by additional specific points made by each of the co‐authors.
